# Chemical Strategies towards the Synthesis of Betulinic Acid and Its More Potent Antiprotozoal Analogues

**DOI:** 10.3390/molecules26041081

**Published:** 2021-02-18

**Authors:** André Barreto Cunha, Ronan Batista, María Ángeles Castro, Jorge Mauricio David

**Affiliations:** 1 Instituto de Química, Universidade Federal da Bahia, Salvador 40170-280, BA, Brazil; andrebarreto@ufba.br; 2 Department of Pharmaceutical Sciences, Pharmaceutical Chemistry Section, CIETUS/IBSAL, Faculty of Pharmacy, University of Salamanca, 37007 Salamanca, Spain; macg@usal.es

**Keywords:** betulinic acid, antiprotozoal activities, triterpene acid derivatives, antiplasmodial activity, antileishmanial activity, antitrypanosomal activity

## Abstract

Betulinic acid (BA, 3β-hydroxy-lup-20(29)-en-28-oic acid) is a pentacyclic triterpene acid present predominantly in *Betula* ssp. (Betulaceae) and is also widely spread in many species belonging to different plant families. BA presents a wide spectrum of remarkable pharmacological properties, such as cytotoxic, anti-HIV, anti-inflammatory, antidiabetic and antimicrobial activities, including antiprotozoal effects. The present review first describes the sources of BA and discusses the chemical strategies to produce this molecule starting from betulin, its natural precursor. Next, the antiprotozoal properties of BA are briefly discussed and the chemical strategies for the synthesis of analogues displaying antiplasmodial, antileishmanial and antitrypanosomal activities are systematically presented. The antiplasmodial activity described for BA was moderate, nevertheless, some C-3 position acylated analogues showed an improvement of this activity and the hybrid models—with artesunic acid—showed the most interesting properties. Some analogues also presented more intense antileishmanial activities compared with BA, and, in addition to these, heterocycles fused to C-2/C-3 positions and amide derivatives were the most promising analogues. Regarding the antitrypanosomal activity, some interesting antitrypanosomal derivatives were prepared by amide formation at the C-28 carboxylic group of the lupane skeleton. Considering that BA can be produced either by isolation of different plant extracts or by chemical transformation of betulin, easily obtained from *Betula* ssp., it could be said that BA is a molecule of great interest as a starting material for the synthesis of novel antiprotozoal agents.

## 1. Introduction

**Figure 1:**
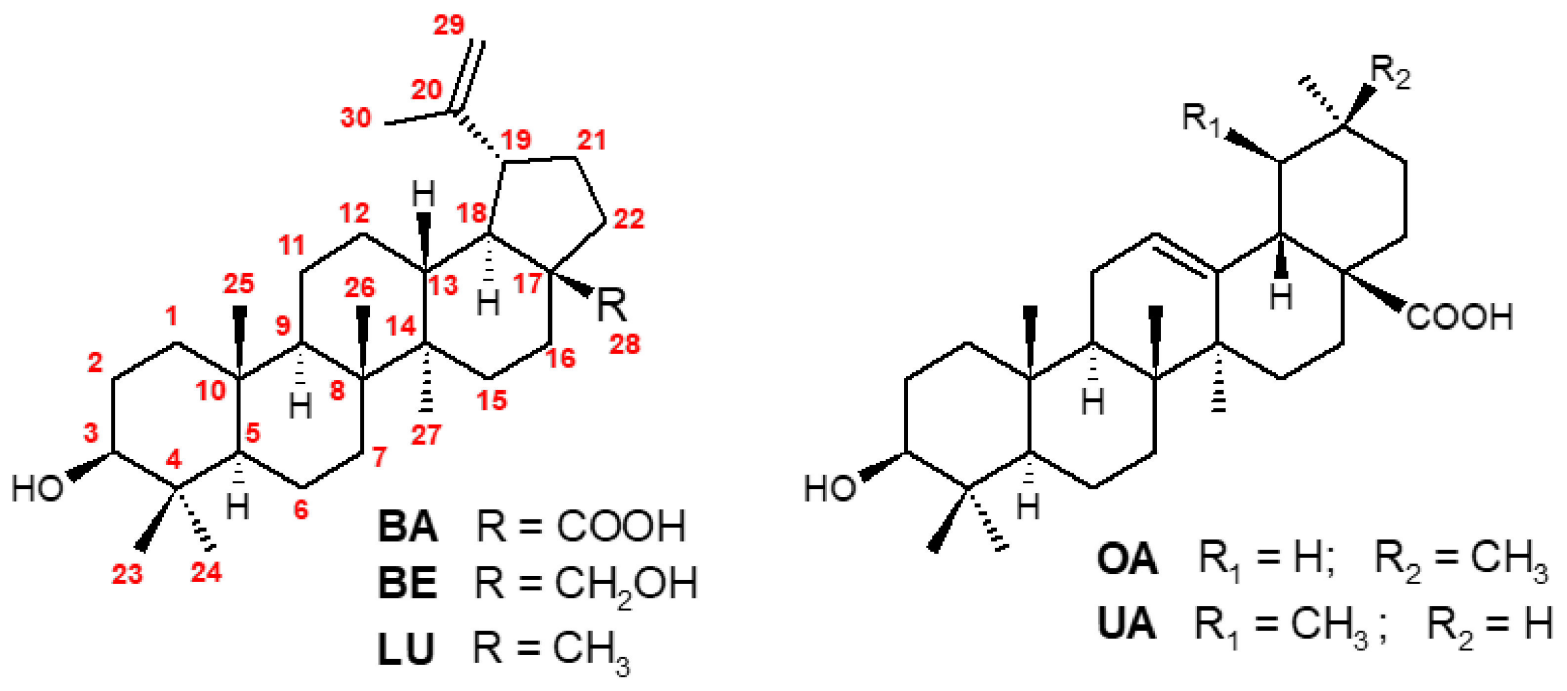
Structures of betulinic acid (BA), betulin (BE), lupeol (LU), oleanolic (OA) and, ursolic (UA) acids.

Betulinic acid (BA), namely 3β-hydroxy-lup-20(29)-en-28-oic acid, is a pentacyclic lupane-type triterpene (Figure 1) which was allegedly isolated for the first time from the bark of *Cornus florida* L. (Cornaceae) in 1939. At that time, its structural elucidation was based on data obtained by comparison with a series of synthetic derivatives [1]. Subsequently, BA was also found in the seeds of *Zizyphus vulgaris* Lam. (var. Spinosus Bunge, Rhamnaceae) [2], in the bark of *Platanus acerifolia* [3] and in the fibrous outer bark of *Syncarpia laurifolia* (currently var. *glomulifera*, Myrtaceae) [4]. For the proper identification of the isolates, the preparation of known chemical derivatives was also necessary. Curiously, it is assumed that BA was found even earlier in other species, but under different trivial names, such as gratiolone (isolated from *Gratiola officinalis*, Schropulariaceae) [5], platanol and/or platanine (from *Platanus* spp.) and an unidentified compound obtained from *Cornus sanguinea* L. and named as “platanoic acid” [3,5,6,7].

The name “Betulinic acid” has been given to this compound because of its prevalence in birch trees, which belong to the genus *Betula*, family Betulaceae, especially *Betula alba*, *B. pubescens*, *B. platyphylla*, *B. maximowicziana* and *B. mandshurica*. Additionally, although this family is the main natural source of BA, this triterpene is spread across many plant species belonging to the families Amaranthaceae, Ancistrocladaceae, Apocynaceae, Asteraceae, Chrysobalanaceae, Convolvuleaceae, Dichapetalaceae, Dilleniaceae, Dioncophyllaceae, Ebenaceae, Ericaceae, Fabaceae, Fagaceae, Lamiaceae, Loganiaceae, Melastomataceae, Moraceae, Myrtaceae, Onagraceae, Ramnaceae, Ranunculaceae, Rhamnaceae, Rosaceae, Rubiaceae, Trochodendraceae, Verbenaceae and Viscaceae [8]. Interestingly, this triterpene can be extracted in large quantities from *Akania lucens* Hook f. (Akaniaceae), *Nerium oleander* L. (Apocynaceae), *Avicennia marina* L. (Verbenaceae), *Lemaireocereus* spp. (Cactaceae), *Arbutus menziesii* Pursh. and *Arctostaphylos uva-ursi* (Ericaceae), *Lavandula angustifolia* var. *officinalis* (Lamiaceae), *Nuytsia floribunda* R. Br. (Loranthaceae), *Tectona grandis* L. f. (Verbenaceae), *Davilla rugosa* and other species of Dilleniaceae family (*Dillenia*, *Wormia* and *Acrotrema*). Thus, BA may be considered as a chemotaxonomic marker for these families/genera [9,10,11]. This compound seems to be biosynthesized from lupeol (LU, Figure 1) by the cytochrome CYP716A12, which has been characterized as a multifunctional enzyme showing lupeol 28-oxidase activities. The same cytochrome is responsible for the methyl C-28 oxidation of β- and α-amirins, providing, in addition to BA, the other common triterpenes olenanolic (OA) and ursolic (UA) acids (Figure 1) [12,13].

Only in 1976 the compound’s pharmacological importance begin to be evidenced. A study employing plant extracts containing BA exhibited high cytotoxicity and selectivity against lymphocytic leukemia P388 cells [14]. Sequentially, BA was identified as a melanoma-specific cytotoxic compound given that in vivo studies showed tumor growth inhibition without toxicity [15]. Since then, various studies with BA and derivatives as antitumor agents have been published [16,17]. Recent investigations have shown that the presence of the carboxyl function at C-28 is required for the cytotoxicity. This conclusion is supported in the fact of BA derivatives are usually significantly more potent than those derived from betulin (BE) (Figure 1) [18]. BA also shows potential as an anti-HIV agent, many of its derivatives, especially hybrids compounds, are highly effective against HIV [19,20]. Compared to azidothymidine (AZT), specific hybrid derivatives presented more potent or equipotent anti-HIV activities but displayed less cytotoxicity [21]. Additionally, numerous studies have been published describing a broad spectrum of other remarkable pharmacological properties for BA against fungi, bacteria, protozoa, diabetes, and inflammatory disorders [22,23,24]. Among all these potential applications for BA and its analogues, we have focused this review on their antiprotozoal activity, and particularly, on their application to tropical diseases transmitted by vectors.

Vector-borne tropical diseases are disorders that are prevalent in tropical and subtropical regions since the cold season in temperate climates often controls the insect populations (vectors) by forcing their hibernation. Among the most impactful tropical diseases, malaria, leishmaniases and trypanosomiases are globally widespread, with potentially harmful consequences if left untreated [25,26].

Malaria is a life-threatening protozoan disease caused by five *Plasmodium* species (*P. falciparum*, *P. vivax*, *P. knowlesi*, *P. ovale* and *P. malariae*) that are transmitted to people through the bites of infected female *Anopheles* mosquitoes. *P. falciparum* and *P. vivax* account for more than 95% of all human malaria infections and therefore represent a great threat and serious challenge to public health. In 2019, with an estimated 229 million cases and 409,000 deaths, nearly half of the world’s population was at risk of malaria. Most cases and deaths occur in sub-Saharan Africa and more severely affect children under 5 years old [27].

In turn, the leishmaniases are a group of diseases caused by protozoan parasites from more than 20 *Leishmania* species. These parasites are transmitted to humans by the bite of an infected female phlebotomine sandfly, and then three main forms of the disease may arise: cutaneous leishmaniasis (CL), the most common form; visceral leishmaniasis (VL), also known as kala-azar, the most severe form; and mucocutaneous leishmaniasis (MCL), the most disabling form of the disease. More than 1 billion people live in areas endemic for leishmaniasis and are at risk of infection. An estimated 30,000 new cases of VL and more than 1 million new cases of CL occur annually [28,29].

Finally, human trypanosomiasis comprises African trypanosomiasis and Chagas disease which are caused by protozoan parasites of the genus *Trypanosoma*. African trypanosomiasis is caused by either *T. brucei gambiense* or *T. brucei rhodesiense* and threatens some 65 million people in sub-Saharan Africa, especially in rural areas and populations disrupted by war or poverty. Alternatively, Chagas disease is caused by *T. cruzi* and is responsible for 21,000 deaths per year, occurring mainly in Latin America [29,30].

Since tropical diseases are not very attractive to pharmaceutical companies for the development of novel drugs [31], few options are available on the market to treat these protozoan disorders. Currently, the most effective antimalarial drugs available are chloroquine and artemisinin derivatives, whereas amphotericin B, paromomycin, miltefosine, pentamidine and sodium stibogluconate are the most important antileishmanial agents. Additionally, pentamidine, benznidazole and nifurtimox are the drugs of choice for the treatment of human trypanosomiasis. The problems related to the high economic and social costs of these drugs, along with their toxic effects and the emergence of drug resistance, point to the urgent need for novel antiprotozoal drugs [32].

This review aims to cover all chemical strategies published so far towards the synthesis of BA and its analogues with potent antiprotozoal activities against *Plasmodium*, *Leishmania* and *Trypanosoma* parasites. 

## 2. Synthesis of BA from Betulin

In view of the highly valuable potential of BA in the development of novel drugs to treat protozoal and other impacting diseases, it is necessary to establish viable sources of this triterpene to allow for its use as a starting material for the preparation of new and improved bioactive analogues. Within this scenario, it is worth mentioning that promising results regarding the production of BA and derivatives by biocatalysis and other biotechnological strategies have been widely reported in the literature [33,34,35] and, therefore, they will not be covered in this review.

To the best of our knowledge, no total synthesis of BA has been reported yet. Although LU, its lupane C-28 methyl co-related triterpene, has been totally synthesized since the 1970s [36,37].

Currently, commercial production of BA substantially depends on traditional phytochemical extraction from birch bark [35], that yields around 0.025% *w/w* from dry material [38]. The structure related to BA, is the triterpene alcohol BE (Figure 1), which is much more abundant in birch trees (up to 34% of the dry barks) [39], but it is biologically less active [38,40] than BA. Therefore, BE could be employed as a starting material to easily prepare BA through semisynthesis, by oxidation of the primary hydroxyl group present at C-28.

The first reports of the synthesis of BA from BE were published in the late 1930s [1,41]. In this study, the authors used routes with a large number of steps and consequently, the overall yield was low.

**Scheme 1:**
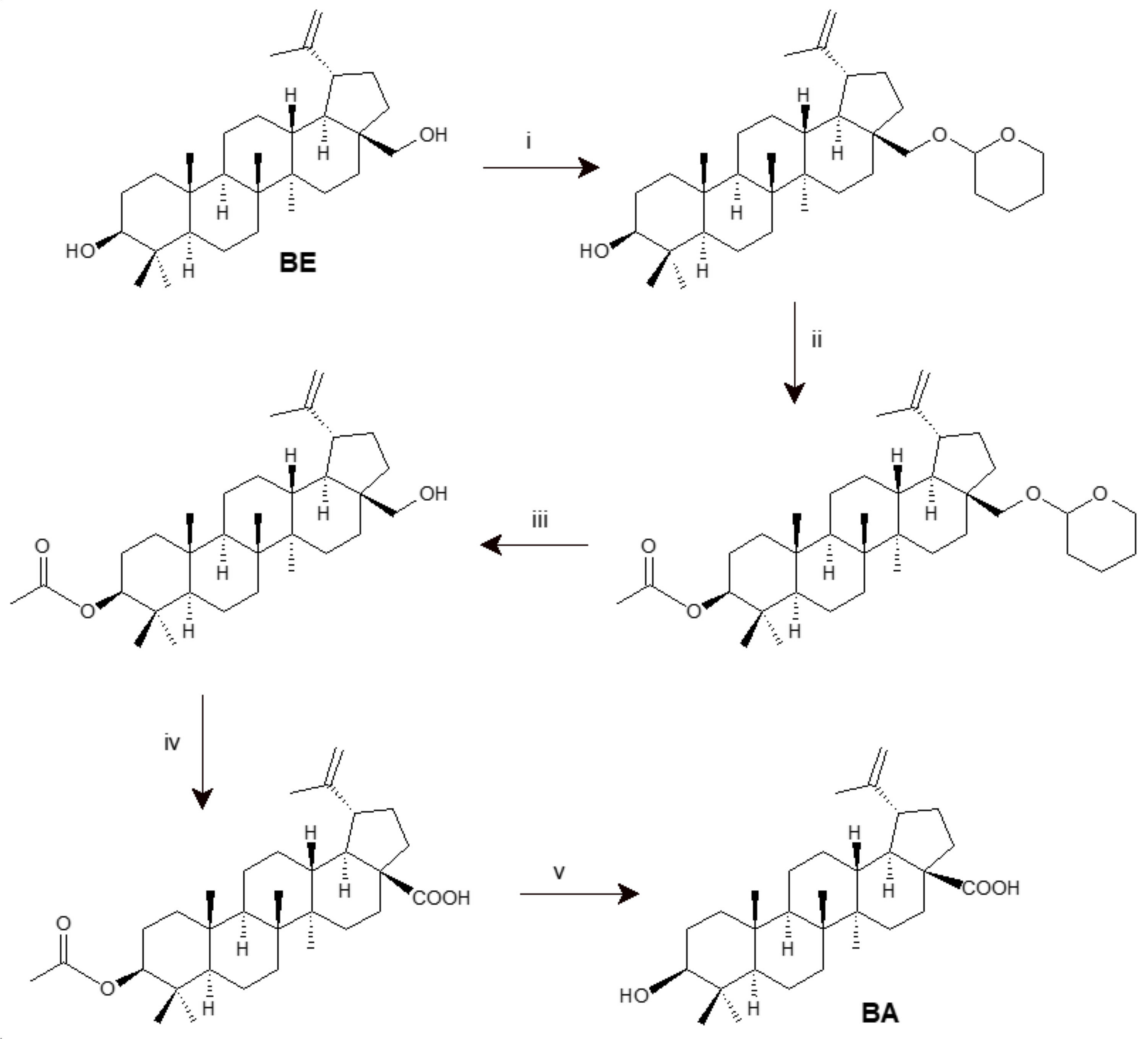
Synthesis of betulinic acid (BA) from betulin (BE) using protecting groups [38]. Reagents and conditions: (**i**) DHP/CH_2_C1_2_/PPTS (95%); (**ii**) Ac_2_O/pyridine (87%); (**iii**) EtOH/PPTS (95%); (**iv**) CrO_3_/H_2_SO_4_/acetone (80%); (**v**) K_2_CO_3_/MeOH/H_2_O (88%).

Decades later, a classical method was reported using protecting groups to selectively mask the C-3 OH group (secondary alcohol) to avoid its isomerization (Scheme 1). In this study, although this classical route involves five steps and uses chemical agents that are to both health and the environment (e.g., CrO_3_), BA could be advantageously produced with high stereoselectivity and a good overall yield [38].

**Scheme 2:**
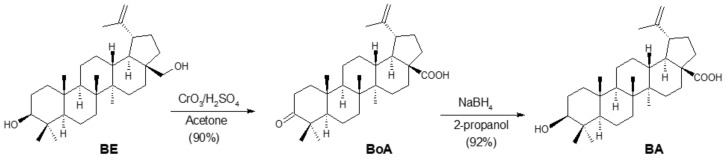
Synthesis of BA from BE through direct Jones oxidation.

However, the most common methodology employed to synthesize BA from BE involves a direct Jones oxidation step (CrO_3_/H_2_SO_4_/acetone) to form first the intermediate betulonic acid (BoA), with a yield of 90%. Then, BoA is reduced with NaBH_4_/2-propanol to obtain, after recrystallization in hot methanol, pure BA. This way, a 3β-isomer yield of 92% is obtained (Scheme 2) [42]. 

**Scheme 3:**
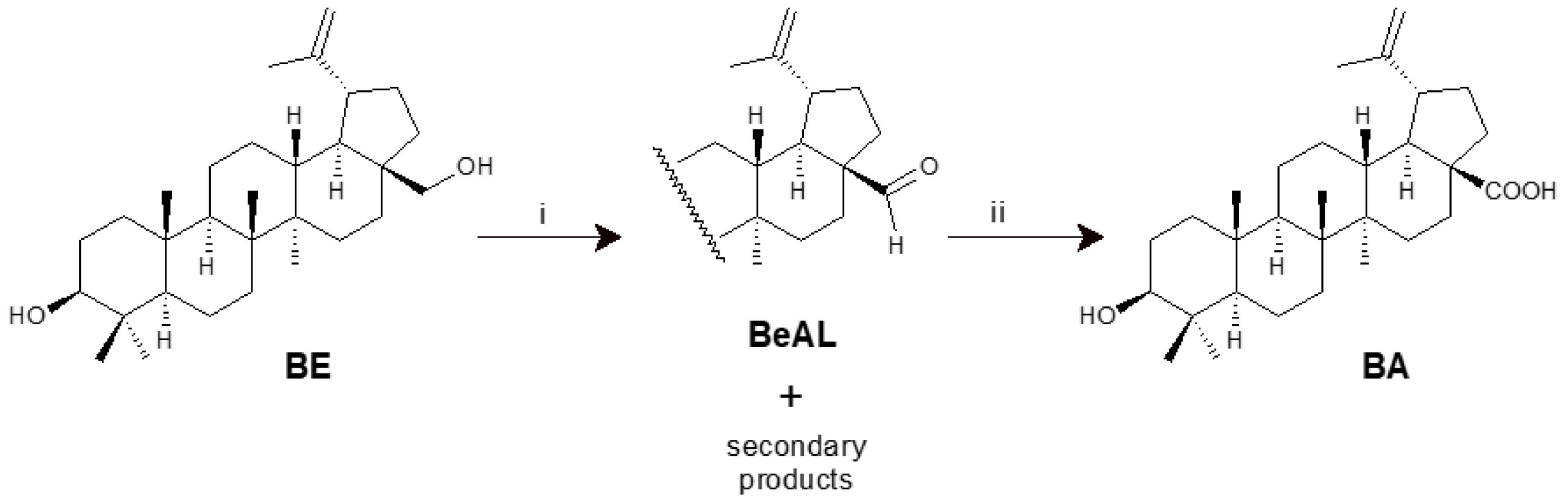
Synthesis of BA from BE via solid-supported CrO_3_ oxidation [43]. Reagents and conditions: (**i**) 2 eq. CrO_3_/SiO_2_ (1:10), toluene, 60 min (84%); (**ii**) 2 eq. KMnO4, acetone, 0 °C, 30 min (~100%).

Recently, a two-step route using solid-supported chromium oxide and potassium permanganate has been suggested to improve BA synthesis [43]. The main step of this approach is the selective oxidation of the primary alcohol function of BE, accomplished with chromic oxide adsorbed on silica gel to obtain BeAL (betulinal) with a reasonable yield (64%). Next, the aldehyde derivative is oxidized to BA by potassium permanganate, resulting in a yield of 85% (Scheme 3). 

**Scheme 4:**
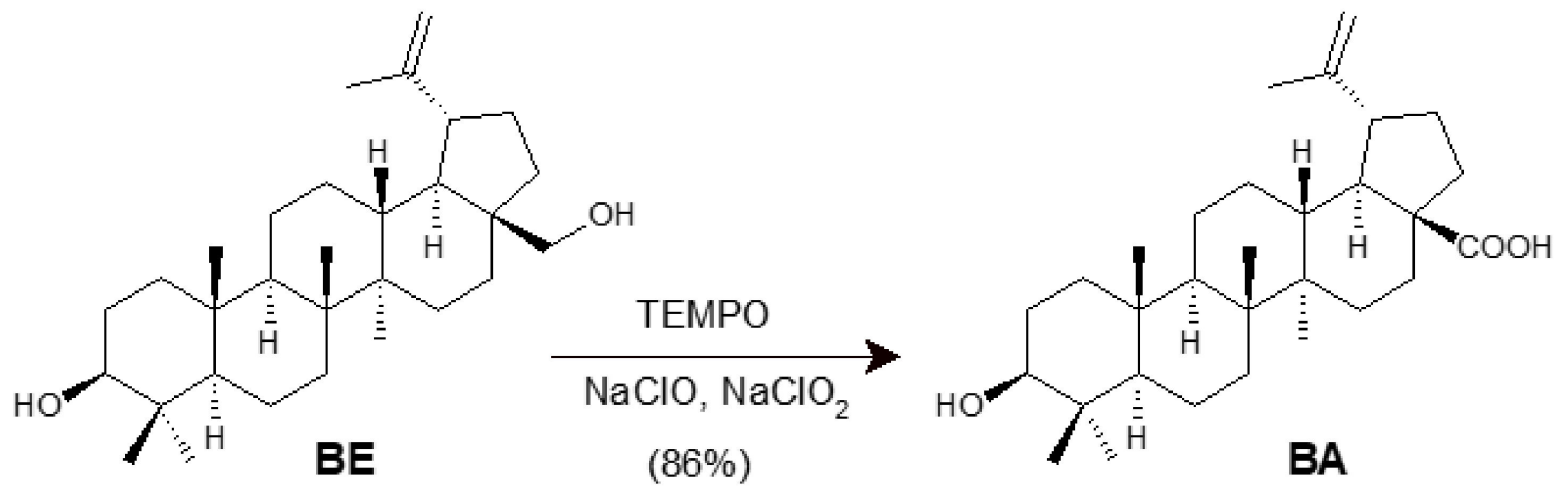
One-step synthesis of BA from BE via 4-acetamido-TEMPO/NaClO/NaClO_2_.

All of the above-mentioned approaches were designed to be small-scale preparations, employing more than one synthetic step with overall moderate to good yields. To overcome these limitations, Csuk and collaborators developed the short one-step route represented in Scheme 4 [40]. The method was based on the catalytic conversion of BE into BA, mediated by 4-acetamide-2,2,6,6-tetramethylpiperidine-1-oxyl (4-acetamide-TEMPO) in a reaction medium containing a mixture of NaClO and NaClO_2_ at 35 °C. It is worth mentioning that the simplicity of the synthetic route is the main advantage resulting from this study, in addition to the use of a cheap starting material and, especially, the possibility of obtaining BA on a larger scale, with a yield of 86% in just one step.

**Scheme 5:**
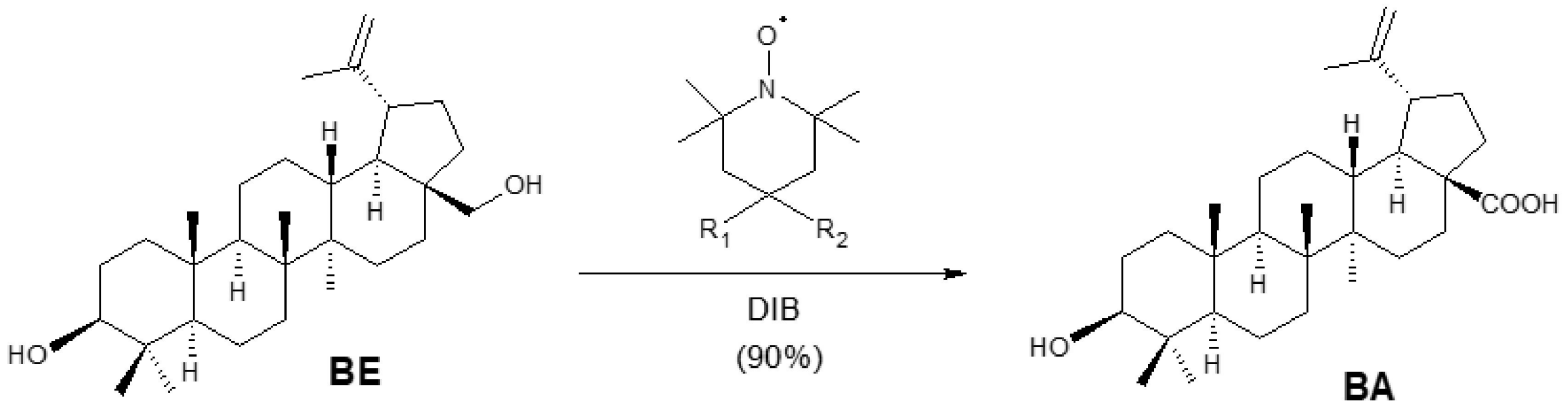
Synthesis of BA from BE via TEMPO-type catalysts/DIB.

In a slightly different one-step method, a patented route also employing TEMPO-type catalysts, the hypervalent iodine reagent diacetoxy-iodobenzene (DIB) was used as the oxidizer [44]. The reaction occurs under mild conditions and is both economically and environmentally friendly, with a yield of up to 90% (Scheme 5).

## 3. Synthesis of Antiprotozoal BA Analogues

**Figure 2:**
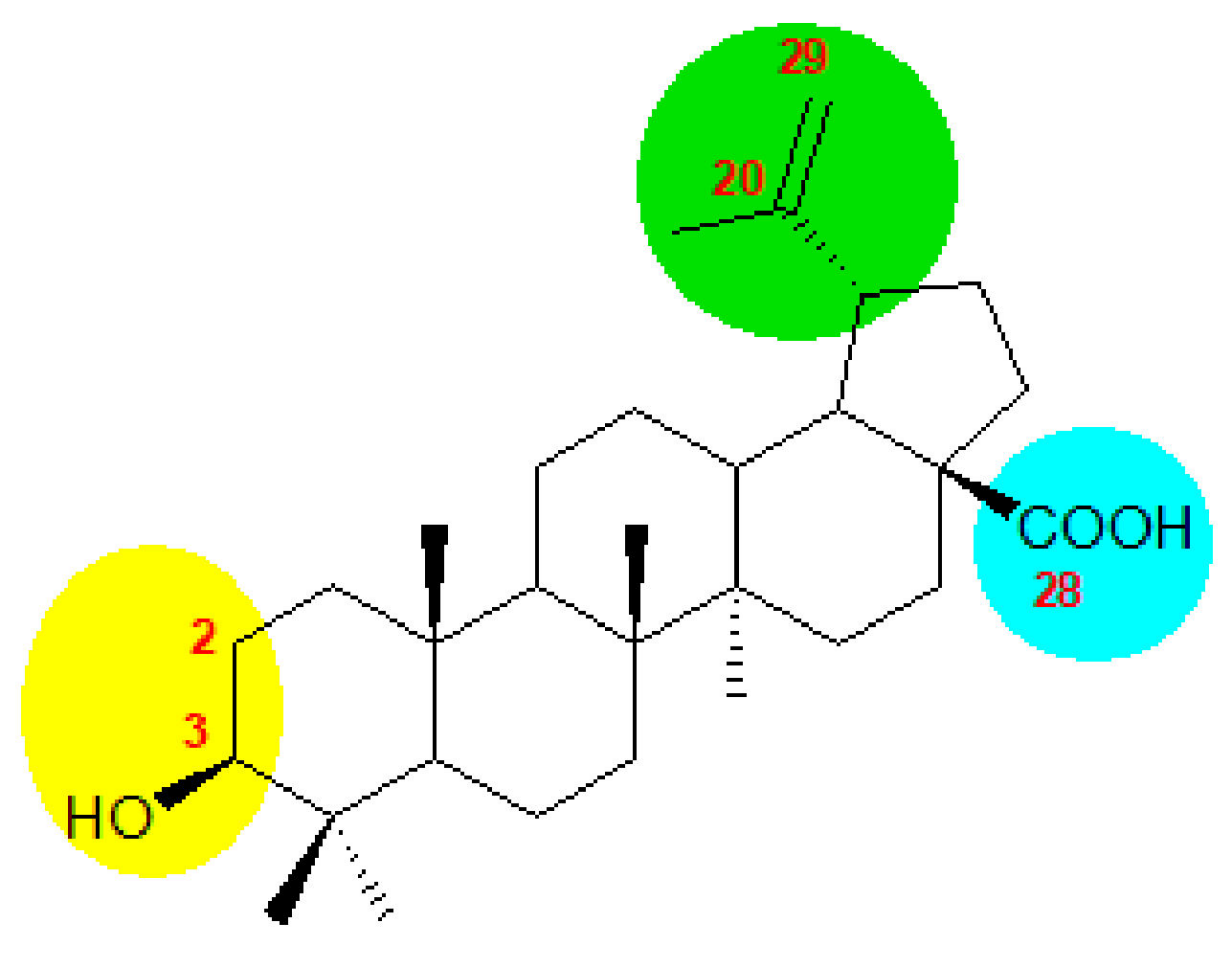
The structural formula of BA and C-2/C-3, C-20/C-29 and C-28 positions amenable to chemical derivatization.

Previous studies have shown a vast range of antimicrobial (including antiprotozoal) properties displayed by triterpenes with lupane skeletons [24,45]. These findings are indicative that the BA could be a promising scaffold for several modifications leading to BA derivatives with more potent antiprotozoal activities. Figure 2 offers an overview of the molecular structure of BA, highlighting positions C-2/C-3, C-20/C-29 and C-28 as those susceptible to chemical derivatization. Thus, in recent years several BA analogues have been designed, synthesized and evaluated against parasites *Plasmodium sp*., *Leishmania sp*. and *Trypanosoma sp*. that are described below.

### 3.1. Antiplasmodial Analogues

The in vitro antiplasmodial properties of BA were reported for the first time by Bringmann and collaborators. This study described that BA exhibited moderate to good antimalarial activity in vitro against asexual erythrocytic stages of the human malaria parasite *P. falciparum* [46].

In a subsequent investigation, the IC_50_ values for BA against chloroquine-resistant (K1) and -sensitive (T9-96) strains of *P. falciparum* were calculated and ranged from 42.9 to 56.7 µM, respectively [47]. In addition, this study also revealed that BA was ineffective at reducing parasitaemia when tested in vivo in a murine malaria model (*P. berghei*), even when the highest dosage of 250 mg/kg/day was used intraperitoneally.

**Figure 3:**
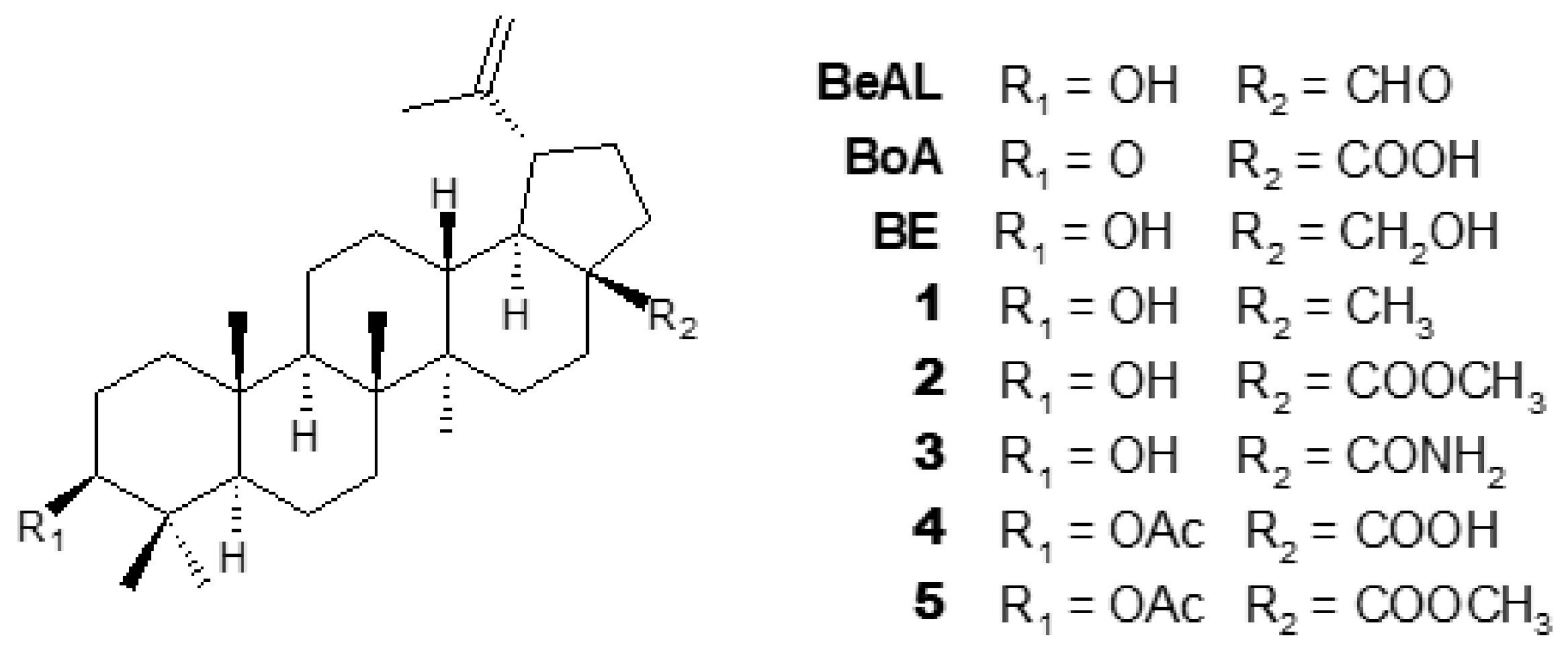
Simple antiplasmodial BA analogues.

Another interesting study showed that erythrocytes preloaded either with BA or its simple analogues BeAL, LU (**1**), BE, BA methyl ester (**2**) and BA amide (**3**) (Figure 3) did not serve as hosts for *P. falciparum* parasites [48]. All the above compounds inhibited *P. falciparum* growth with IC_50_ values in the range of 7–28 µM, and this inhibition correlates well with the extent of membrane curvature changes towards stomatocytes or echinocytes in a concentration-dependent approach. The authors concluded that the antiplasmodial activities of BA and its analogues are clearly related to the incorporation of such compounds into the lipid bilayer of erythrocytes, causing modifications of cholesterol-rich membrane rafts that play an important role in parasite vacuolization. The authors also concluded that this established link between erythrocyte membrane modifications and antiplasmodial activity might suggest a novel target for the development of novel antimalarial drugs [48].

The in vitro evaluation of BA and its simple derivatives BoA, betulinic acid acetate (**4**), BA methyl ester (**2**) and BA methyl ester acetate (**5**) (Figure 3) against chloroquine-resistant *P. falciparum* parasites showed IC_50_ values of 9.89, 10.01, 5.99, 51.58, and 45.79 μM, respectively [49]. In addition, mice infected with *P. berghei* and treated with **4** showed a dose-dependent reduction in parasitemia. These results are in agreement with those later obtained by Domínguez-Carmona [45], who showed that simple esterification of the C-3 hydroxyl group results in an improvement of the antiplasmodial activity.

**Scheme 6:**
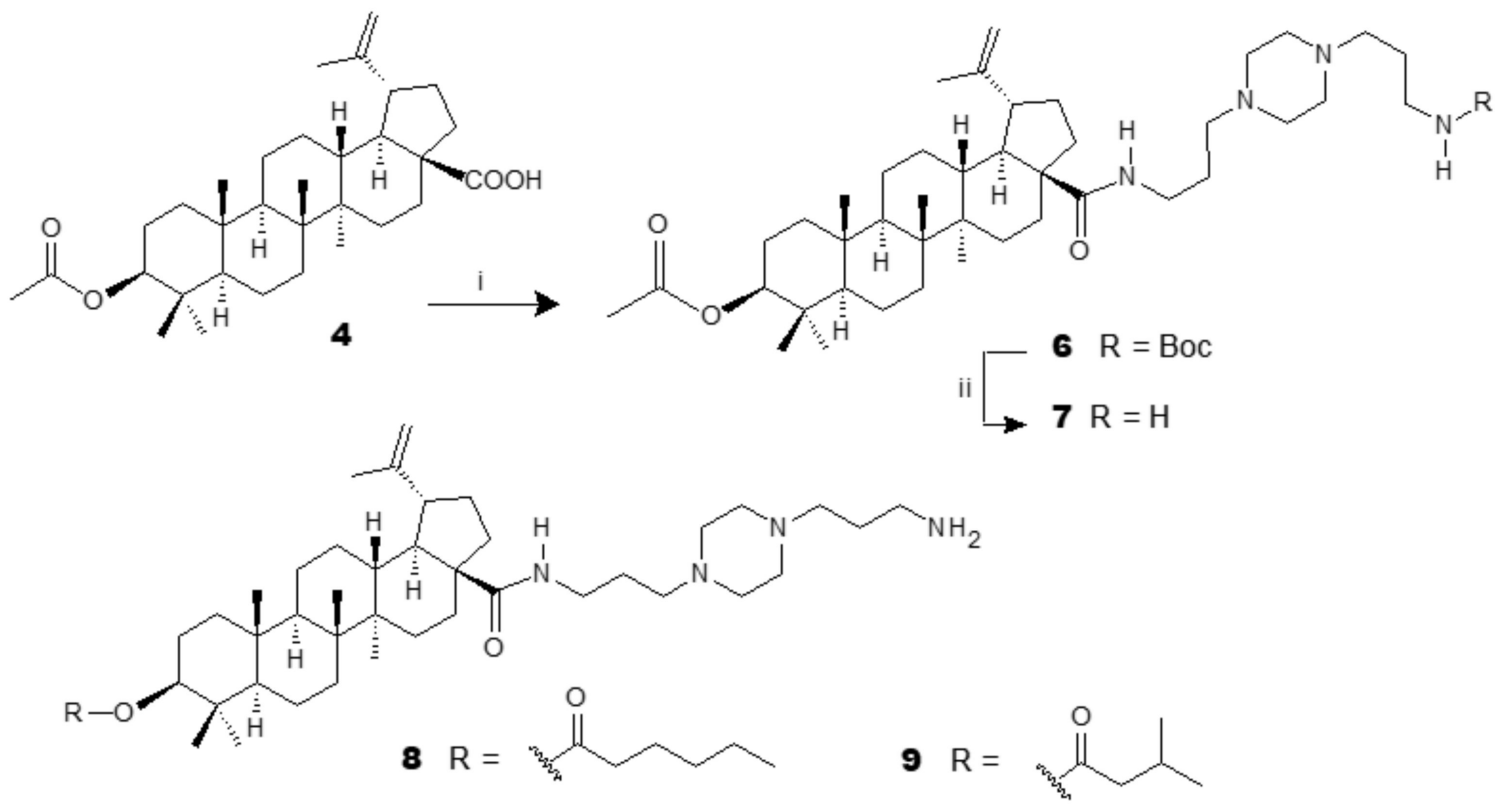
Synthesis of new piperazinyl derivatives of acetyl betulinic acid. Reagents and conditions: (**i**) ClCOCOCl, 0 °C, 3 h; TEA, *N-tert*-butoxycarbonyl-1,4-bis(3-aminopropyl)piperazine, RT, 24 h; (**ii**) TFA 10%/CH_2_Cl_2_, RT, 6 h.

Based on studies that reported that piperazine derivatives possess antimalarial action, new piperazinyl derivatives at the C-28 position of **4** [50] were designed and synthesized as prototypes for new antimalarial compounds against the *P. falciparum* chloroquine-sensitive 3D7 strain (Scheme 6). Among them, compounds **4** (IC_50_ = 4 μM) and **6** (IC_50_ = 5 μM) were found to show good antiplasmodial activity, while **7** was shown to be much more potent (IC_50_ = 220 nM), also presenting a selectivity index = 18. However, subsequent in vivo studies [51] revealed that **7** showed toxicity to mice, and docking studies indicated the putative mechanism of action of **7** in inhibiting PfATP6, a SERCA-type Ca+2-ATPase present in *P. falciparum*, as well as the mammalian SERCA protein with greater affinity, compromising the selectivity expected towards the parasite. 

The study performed by Innocente and collaborators [50] was useful to inspire the synthesis and antiplasmodial assessment of novel BA piperazinyl analogues, the results of which were published some years later [52]. Analogues **8** (IC_50_ = 1 μM) and **9** (IC_50_ = 4 μM) (Scheme 6) displayed good antiplasmodial potency against the *P. falciparum* chloroquine-sensitive 3D7 strain. Moreover, the authors investigated the mechanism of action of **8**, and noted that this derivative led to an increase in cytosolic Ca^2+^ as well as causing a lower inhibition of β-haematin formation than that observed for chloroquine.

**Figure 4:**
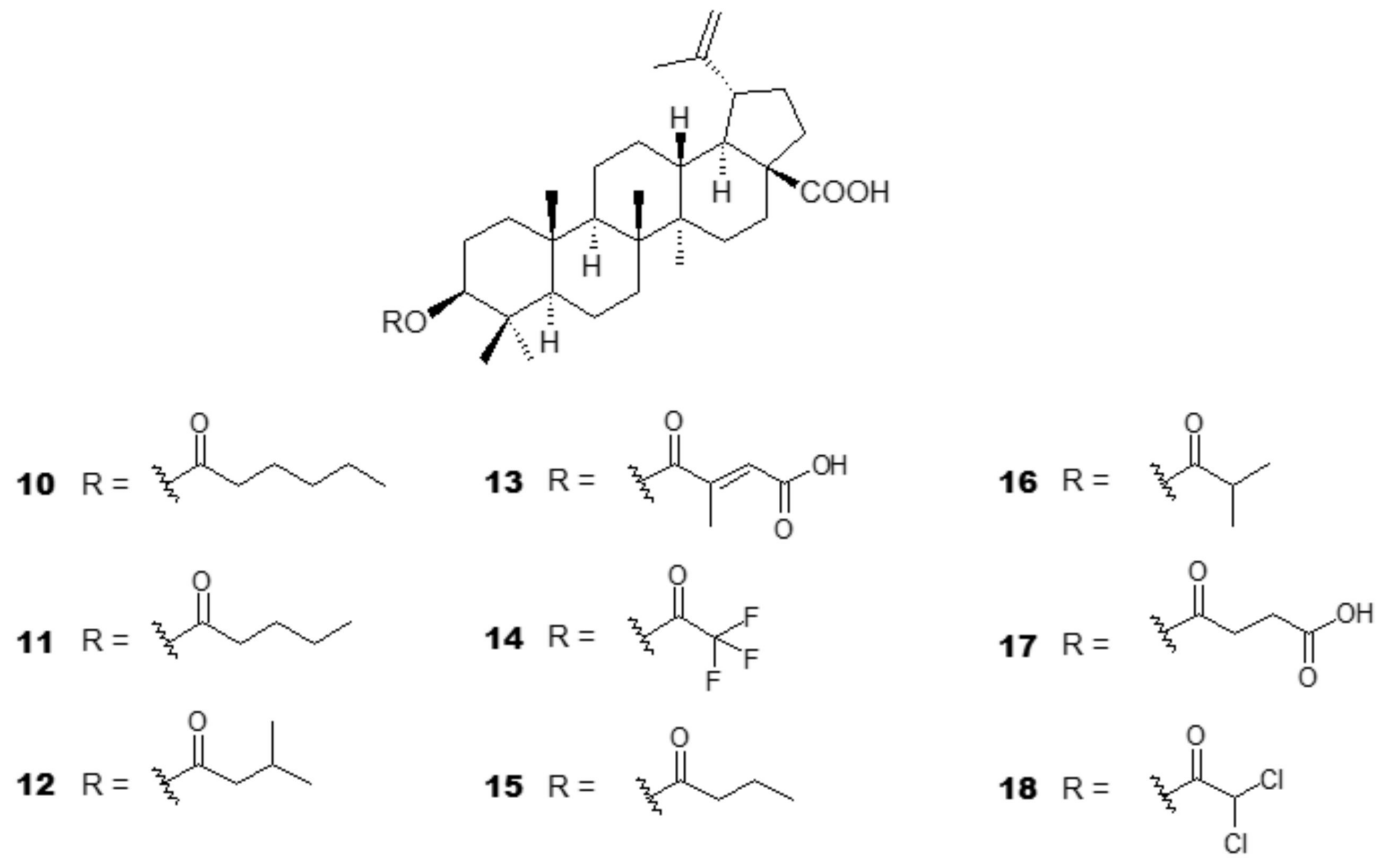
Antiplasmodial BA analogues from esterification at the C-3 OH group.

A new series of BA derivatives **10**–**18** obtained by carboxylic acid esterification at the C-3 OH group was synthesized in an attempt to improve the antimalarial properties of the BA skeleton. Among the nine derivatives prepared (Figure 4), two presenting smaller side chains (**15** and **16**, IC_50_ = 5–8 µM) were found to be two to three times more potent than BA (IC_50_ = 18 µM) against chloroquine-sensitive *P. falciparum* 3D7. Additionally, such analogues were non-cytotoxic towards the mammalian cell line HEK293T [53]. 

In another study, the synthesis of a novel series of BA derivatives was performed by acetic, butyric and isobutyric esterification at the C-3 OH group, as well as by incorporating methoxy and imidazole moieties at the carboxyl at the C-28 position [54]. The antiplasmodial activities of all analogues were assessed against the chloroquine-resistant *P. falciparum* W2 strain. The results permitted the conclusion that structural modifications at C-3 were more promising for increasing the antiplasmodial properties of the BA skeleton, rather than simultaneous modifications at the C-3 and C-28 positions. From all the prepared compounds, the ester derivative 3β-butanoyl betulinic acid (**15**) was shown to be the most potent (IC_50_ = 3.4 µM) and it also did not present cytotoxicity against VERO nor HepG2 cells (CC_50_ > 400 µM), thus displaying a selectivity index higher than 117. Docking studies indicated a possible interaction of **15** with the *Plasmodium* protease PfSUB1. Moreover, additional data suggest that the main target of **15** on *Plasmodium* might be related to other molecules and processes on the ring stage.

**Figure 5:**
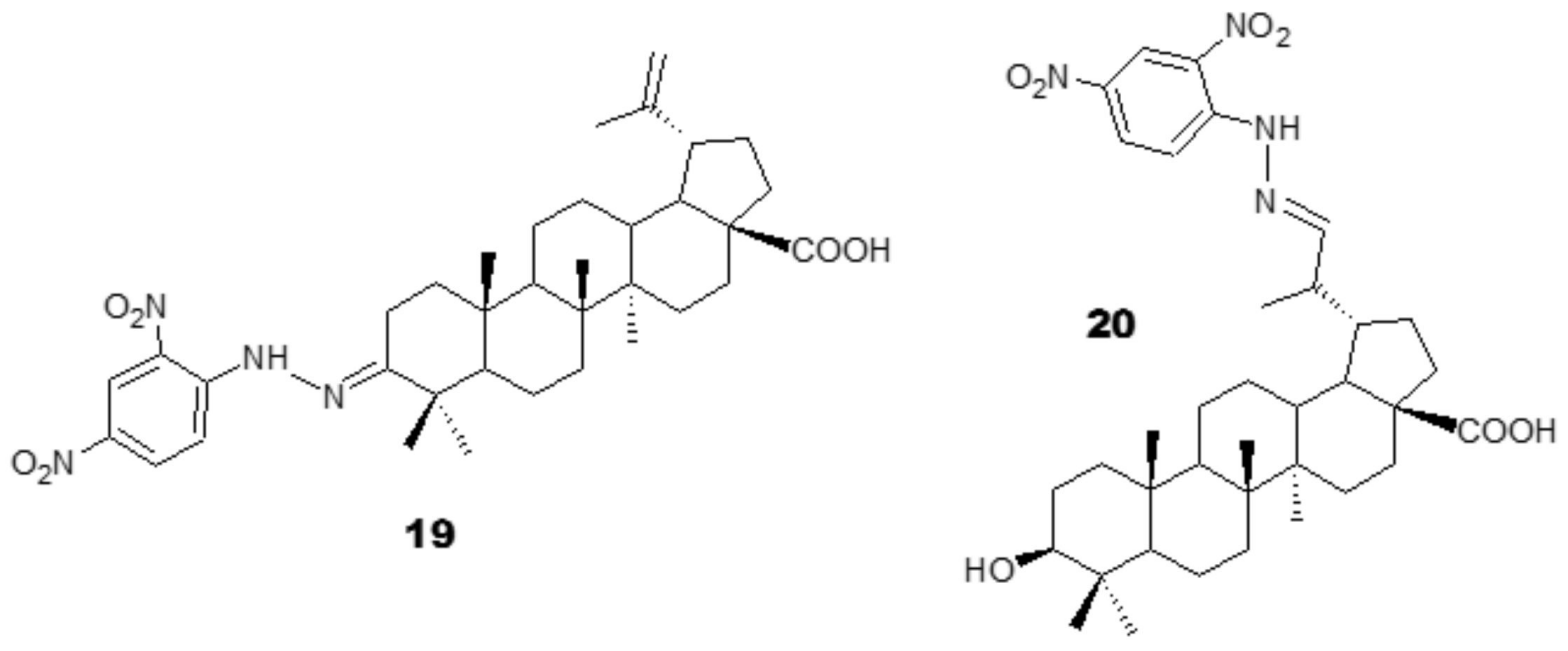
2,4-Dinitrophenyl-hydrazone-BA analogues with moderate antiplasmodial activity.

In a different chemical approach, nine 2,4-dinitrophenyl-hydrazono-BA analogues were prepared [55], but only compounds **19** (IC_50_ = 15.3 µM) and **20** (IC_50_ = 10.2 µM) (Figure 5) showed better antiplasmodial activity than BA (IC_50_ = 38.8 µM) against the chloroquine-resistant *P. falciparum* W2 strain. 

**Scheme 7:**
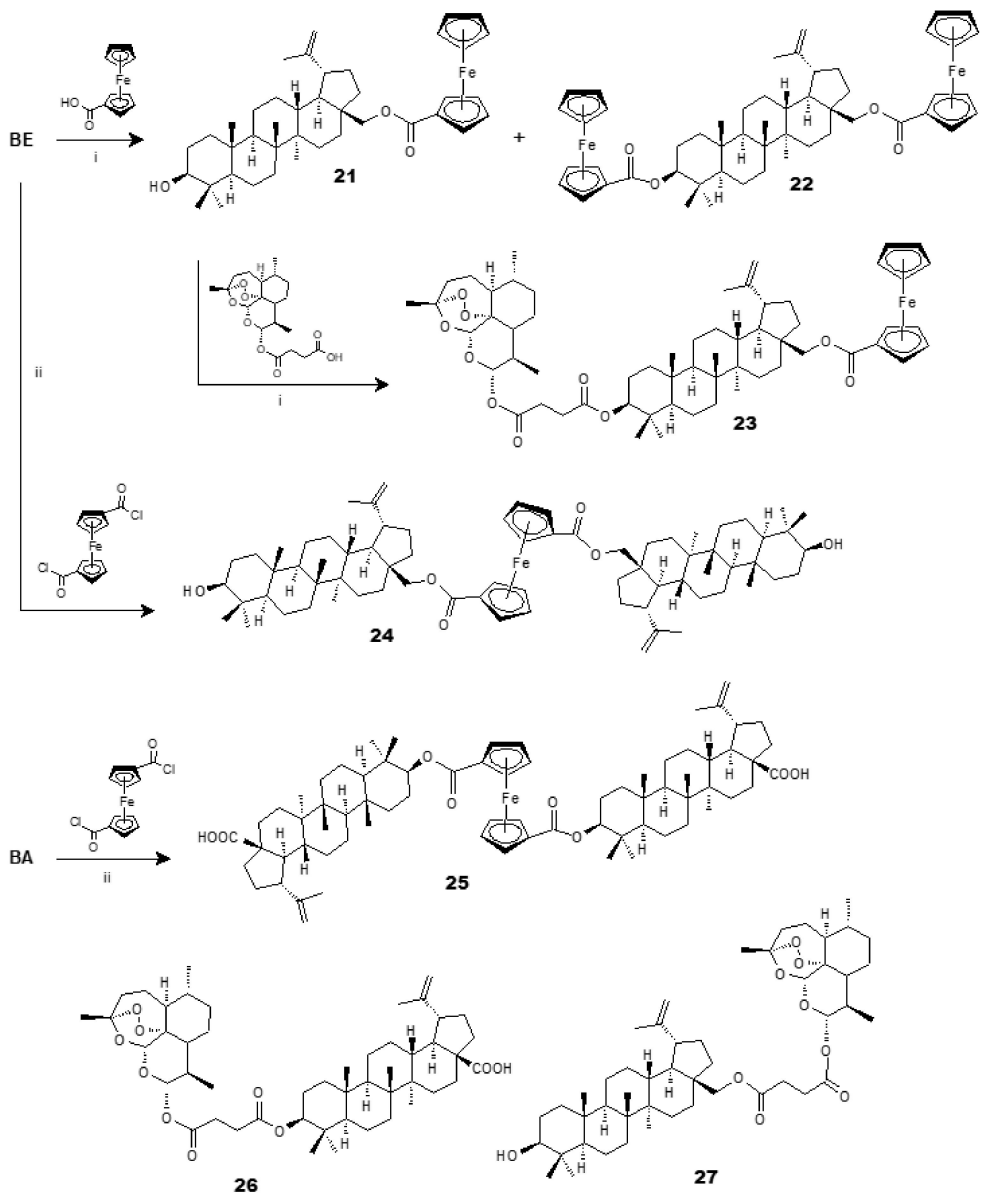
Synthetic routes to BA/BE-derived dimers and hybrids **21**–**25**. Reagents and conditions: (**i**) DCC, DMAP, CH_2_Cl_2_, 0 °C→rt; (**ii**) DMAP, CH_2_Cl_2_, 0 °C→rt.

Inspired by the highly active antimalarial compound ferroquine and by the previously prepared artemisinin–ferrocene hybrid, a series of BA/BE based dimer and hybrid compounds carrying ferrocene and/or artesunic acid moieties were designed and synthesized (Scheme 7), to include the ferrocene moiety into the new molecules as a linker or a subunit [56]. The antiplasmodial activity of all newly synthesized compounds **21–25** as well as the previously synthesized hybrids **26** and **27,** were evaluated against *P. falciparum* 3D7 strain. Compounds **23** (IC_50_ = 1.49 µM), **26** (IC_50_ = 0.26 µM) and **27** (IC_50_ = 0.09 µM), which are hybrids of BA and artesunic acid, were found to be the most active antiplasmodial analogues.

All the above described results indicated that BA is a weak antimalarial agent, and consequently, it is not suitable to be employed as a drug for this disease. However, the improvement in the antiplasmodial activity that was found for some of its derivatives makes BA a good starting material to design and prepare new analogues that are able to reach and even improve the potency of the commercially available antimalarial drugs. In this sense, new chemical approaches may explore other functionalities at the C-3 and/or C-28 positions in order to synthesize more potent antiplasmodial analogues. Within this scenario, hybridization with other antimalarial drugs seems to be a promising synthetic strategy.

### 3.2. Antileishmanial Analogues

Betulinal (BeAL) was the first naturally occurring BA-structurally related compound with antileishmanial properties to be reported in the literature. It showed a weak activity in vitro, reducing *Leishmania amazonensis* amastigotes infection by 88% at 136 µM, but this high dose was also significantly toxic to peritoneal macrophages [57]. However, the analogue 20(29)-dihydrobetulinic acid (DHBA) was described as being capable of strongly inhibiting the growth of *L. donovani* promastigotes (IC_50_ = 2.6 µM) and amastigotes (IC_50_ = 4.1 µM) at lower concentrations [58]. Furthermore, DHBA was also shown to be a dual inhibitor of DNA topoisomerases I and II and acts by preventing the formation of enzyme-DNA binary complex, thus inducing apoptosis.

Later, in a screening study aiming to investigate the in vitro leishmanicidal activity of 46 scarce natural products, the weak antileishmanial effect of BA (IC_50_ = 87.5 µM) was then first evidenced against *L. major* promastigotes [59]. Similarly, betulinic acid acetate (**4**, IC_50_ = 44.9 µM) and BoA (IC_50_ = 51.2 µM) derivatives were also both weakly active against *L. amazonensis* [45].

**Scheme 8:**
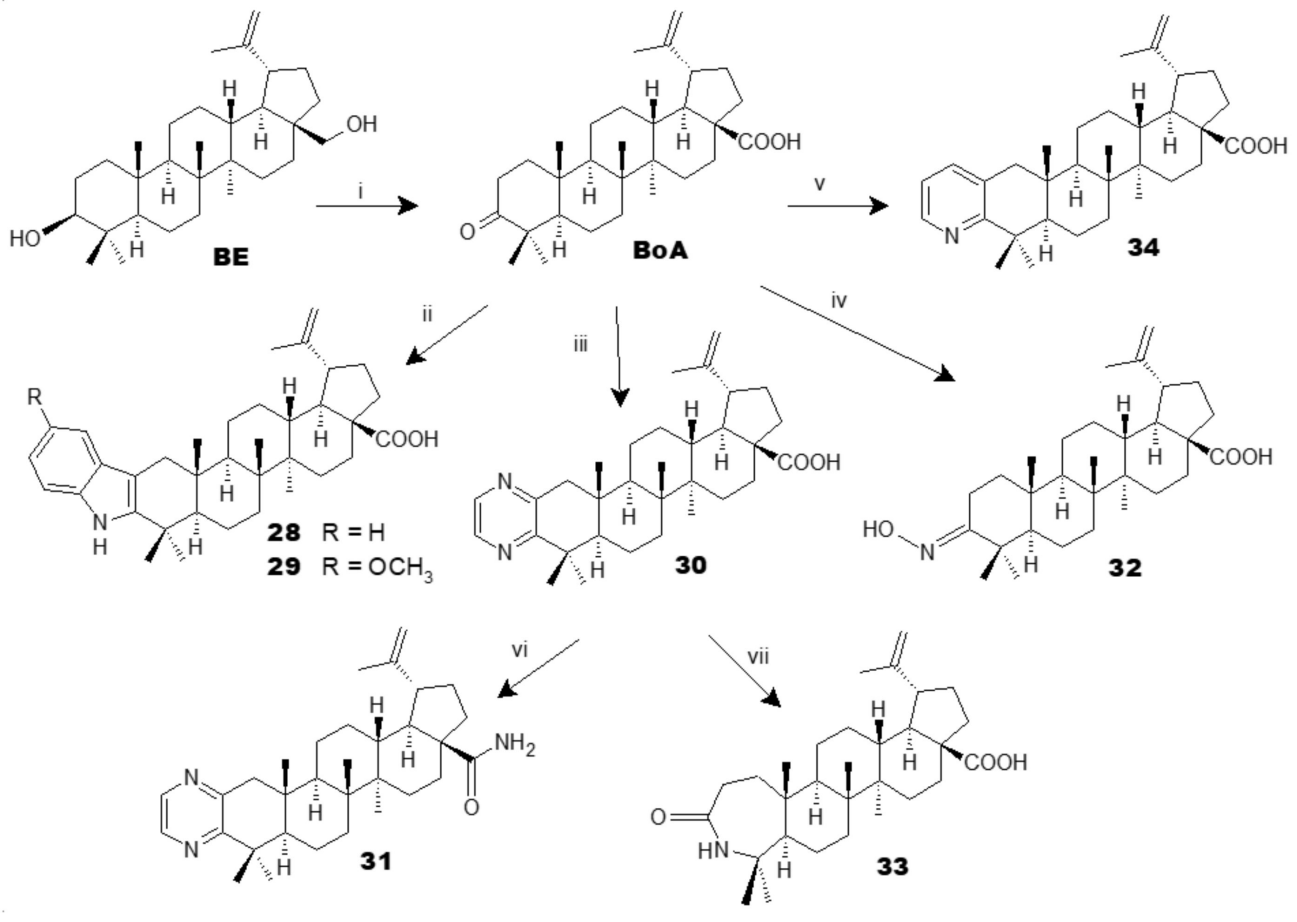
Synthesis of heterocyclic BA analogues [60]. Reagents and conditions: (**i**) Jones oxidation, Na_2_Cr_2_O_7_, H_2_SO_4_, H_2_O, acetone, rt, 21 h (44%); (**ii**) appropriate phenylhydrazine hydrochloride, HOAc, reflux, 3 h, (21–42%); (**iii**) ethylenediamine, sulfur, morpholine, reflux, 21 h (68%); (**iv**) NH_2_OH/HCl, pyridine, MeOH, reflux, 16 h (84%); (**v**) propargylamine, Cu(I)Cl, EtOH, reflux, 17 h (11%); (**vi**) 1. oxalyl chloride, DCM, rt, 3 h; 2. aqueous ammonia, DCM, rt, 1 h, (~100%); (**vii**) TFAA, DCM, rt, 20 h, (33%); DCM ¼ dichloromethane, TFAA ¼ trifluoroacetic anhydride.

Heterocyclic analogues **28**–**34** were designed and synthesized from BE, employing BoA as a versatile intermediate. The employment of the cheap BE instead of BA is an advantage of this route. Different heterocycles were fused to the 2,3-position of the lupane skeleton, including isoxazole, pyrazine, pyridine, indole and pyrazole groups, while the carboxyl group at the C-28 position was also transformed into an amide derivative (Scheme 8). Among all compounds tested, the analogues **30** (IC_50_ = 13.2 µM) and **33** (IC_50_ = 4.3 µM) were found to display the best antileishmanial activity against axenic amastigotes of *L. donovani*, with derivative **33** being both the most potent and selective (SI = 12.9) antileishmanial analogue of all those reported in this study [60].

**Scheme 9:**
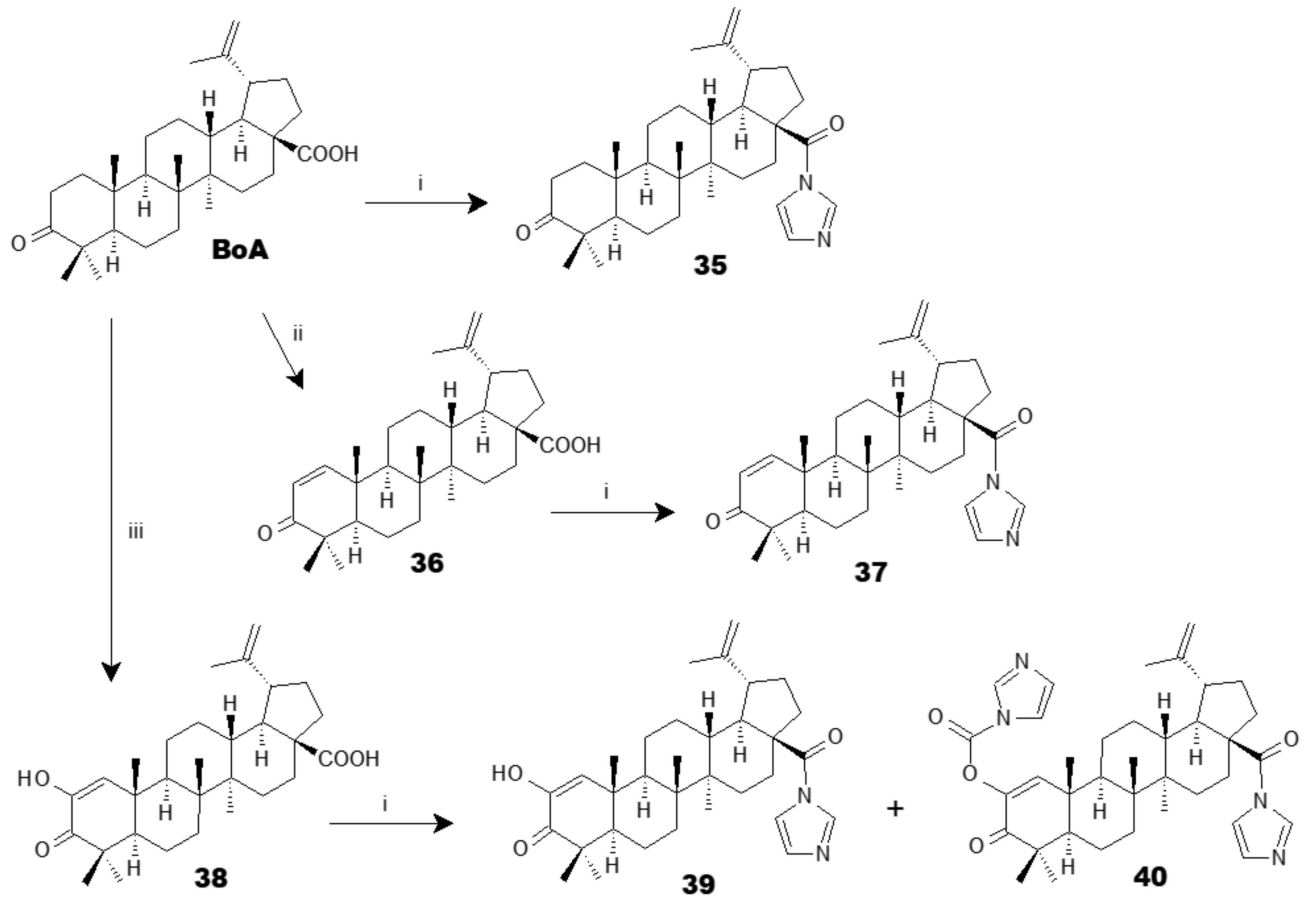
Synthesis of imidazole BA analogues [61]. Reagents and conditions: (**i**) CDI, dry THF, N_2_, reflux, 8–9 h; (**ii**) DDQ, dioxane, N_2_, reflux, 15 h; (**iii**) O_2_, *t*-BuOK, *t*-BuOH, 40ºC, 2 h.

The new imidazole carbamate and *N*-acylimidazole derivatives **35**–**40** were synthesized from BoA (Scheme 9) and firstly, they were screened for in vitro cytotoxicity activity against human cancer cell lines [61]. Later, these imidazole analogues were also assessed against *L. infantum* promastigotes [62]. Compound **40** (IC_50_ = 25.8 µM), which has an additional imidazole carbamate at the C-2 position, was shown to be the most active analogue, and its antileishmanial activity was synergistically increased when **40** was associated with miltefosine, the medication used to treat leishmaniasis.

Antileishmanial studies employing BA derivatives are still in early stages. Even though only a few results have been obtained up to the present time, they suggest that other known BA analogues may display interesting antileishmanial profiles and, thus, they also deserve to be assayed in vitro and in vivo against *Leishmania sp.* parasites, including in combination with traditional antileishmanial drugs. Furthermore, taking into account that heterocyclic betulin derivatives displayed antileishmanial activities with GI_50_ values between 8.9–30.0 µM [63], novel heterocyclic BA analogues are worthy of attention for synthesis and antileishmanial investigation. 

### 3.3. Antitrypanosomal Analogues

BA shows weak antitrypanosomal properties, first revealed when this triterpene was isolated from a *Strychnos spinose* Lam. lipophilic leaf extract showing antitrypanosomal activity. This plant of the Loganiaceae family is traditionally used to treat African trypanosomiasis. The BA isolated was assayed against bloodstream forms of *Trypanosoma brucei* and showed IC_50_ = 32.6 µM (SI = 1.3) [64]. Subsequently, BA was also assayed against epimastigote forms of *T. cruzi* Tulahuen strain, and again BA was weakly active (IC_50_ = 50.0 µM) [45].

**Figure 6:**
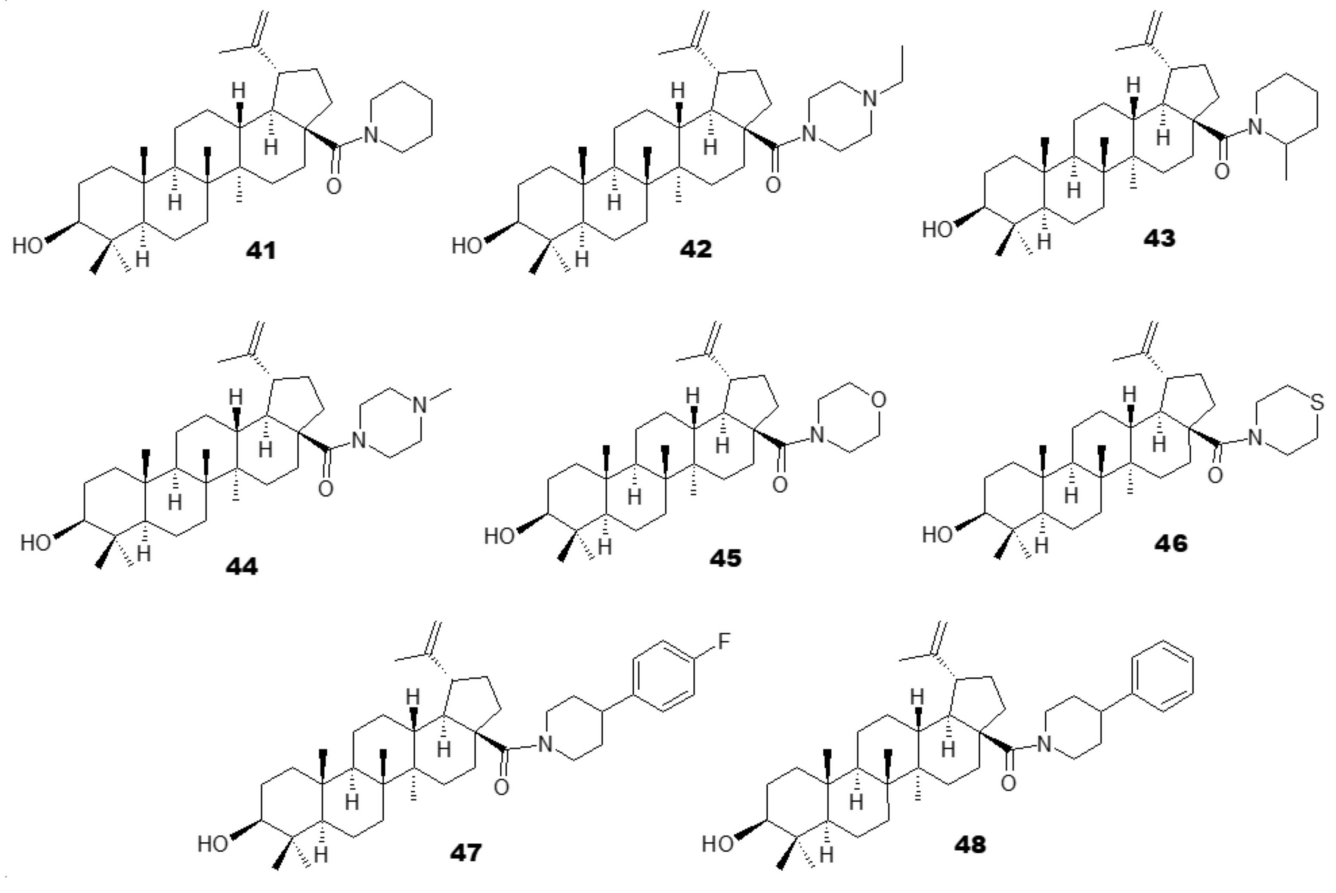
Amide BA analogues with antitrypanosomal activity.

These findings indicated that BA could be considered a good lead for the design and synthesis of more potent and selective antitrypanosomal analogues [65]. Previous studies have suggested that changes at the C-28 carboxyl group could lead to analogues with enhanced antiprotozoal activities when compared to BA. Thus, new amide analogues **41**–**48** (Figure 6) were prepared and assayed against trypomastigotes forms of *T. cruzi*. The most potent antitrypanosomal effects were observed for **45** (IC_50_ = 1.8 µM; SI = 17.3), **46** (IC_50_ = 5.4 µM; SI = 5.3) and **48** (IC_50_ = 5.0 µM; SI = 10.7), while BA displayed an IC_50_ value of 19.5 µM and SI = 1. In this study, the authors identified **45** as a selective anti-*T. cruzi* agent, and additional experimental data showed this analogue destroys parasite cells by necrotic death and also exhibits synergistic activity when used in combination with benznidazole, the antiparasitic drug commonly employed in the treatment of Chagas disease [65]. 

These initial results reveal promising antitrypanosomal properties of BA analogues and open the door to unexplored opportunities for assaying numerous other BA derivatives that have already been synthesized [66,67] against *Trypanosoma sp.* parasites. Moreover, new chemical approaches aiming at incorporating triphenylphosphonium groups into the BA skeleton [68] may improve not only physical properties, but also the mechanism of action of BA analogues, leading to more potent antitrypanosomal compounds. 

## 4. Concluding Remarks and Perspectives

BA is a compound that is easily obtained from natural sources or by the chemical transformation of biosynthetically related triterpenes. Its total synthesis is still not commercially possible, but semisynthesis from BE has yielded good results. In addition, besides the already reported chemical procedures used to obtain BA and its derivatives from natural precursors, some promising biotechnological developments have also been achieved in the same direction by employing biocatalysis of BE and BA by microorganisms [13,34,69,70,71] and plant cells [72].

BA and its derivatives present a range of biological activities, especially anticancer and anti-HIV properties. However, the antiprotozoal activity is also very interesting, particularly when dealing with vector-borne tropical diseases such as malaria, leishmaniases and trypanosomiases, which have a high prevalence in tropical and subtropical regions. The antiplasmodial activity described for BA was moderate, nevertheless some analogues that were acylated at the C-3 position showed an improvement of this activity and the hybrid models with artesunic acid showed the most interesting properties. Similarly, several analogues also presented more intense antileishmanial activities than BA, and heterocycles fused to the C-2/C-3 positions and amide derivatives were the most promising analogues. Regarding the antitrypanosomal activity and besides the weak activity of BA, some interesting antitrypanosomal analogues were also prepared by amide formation at the C-28 carboxylic group of the lupane skeleton.

To date, most of the chemical transformations performed on the BA structure deal with esterification of the C-3 hydroxyl group and derivatization of the C-28 carboxylic group, however, another functional group present in BA that is susceptible to transformation is the double bond at C-20/C-29. This has not been well explored, and no robust studies aimed at synthesizing BA antiparasitic analogs from structural modifications at these positions have been found in the literature yet. Just one recently published study suggested that BA bromination at the C-20/C-29 positions may generate more potent antileishmanial analogues [73]. In our opinion, further studies would be of interest to obtain new derivatives by transformation not only of the double bond itself, but also of the allylic positions. Its evaluation as an antiprotozoal would allow for gaining more in-depth knowledge regarding the structure–activity relationships of BA.

It is well known that molecular conjugation or hybridization can yield compounds with improved pharmacokinetic and pharmacological properties, and even overcome resistance in comparison with their precursors [74,75,76]. In this sense, taking into account that hybridization of BA with artesunic acid gave good results, it will be worth exploring the application of this strategy to other known antimalarial, antileishmanial and antitrypanosomal agents.

BA can be produced either by isolation of different plant extracts or by the chemical transformation of betulin; easily obtained from Betula ssp. Consequently, it can be said that BA could be a lead molecule of great interest for further research into obtaining new commercial products, with the potential to be used as antiprotozoal agents, including for the treatment of neglected tropical diseases.
